# Improved Early Urinary Continence After Robot-Assisted Radical Prostatectomy Using a Modified Vesicourethral Anastomosis with Posterior Musculofascial Reconstruction: A Prospective Comparative Study

**DOI:** 10.3390/jcm15082933

**Published:** 2026-04-12

**Authors:** Paolo Pietro Suraci, Manfredi Bruno Sequi, Fabio Maria Valenzi, Yazan Al Salhi, Onofrio Antonio Rera, Michele Di Dio, Damiano Graziani, Giorgio Martino, Giuseppe Candita, Filippo Gianfrancesco, Paolo Benanti, Luca Erra, Giovanni Di Gregorio, Battista Lanzillotta, Antonio Carbone, Antonio Luigi Pastore, Andrea Fuschi

**Affiliations:** 1 Urology Unit, Department of Medico-Surgical Sciences and Biotechnologies, Faculty of Pharmacy and Medicine, Sapienza University of Rome, Via Franco Faggiana 1668, 04100 Latina, Italy; 2 Department of Surgical Sciences, University of Rome “Tor Vergata”, Via Montpellier 1, 00133 Rome, Italy; 3 Division of Urology, Department of Surgery, Annunziata Hospital, Via Felice Migliori 1, 87100 Cosenza, Italy

**Keywords:** RARP, continence, PMFR, reconstruction

## Abstract

**Introduction:** Post-prostatectomy incontinence (PPI) remains a major functional concern after robot-assisted radical prostatectomy (RARP). Posterior musculofascial reconstruction (PMFR) has been shown to facilitate early urinary continence (EUC), but variations in technique may further improve outcomes. We evaluated whether a modified vesicourethral anastomosis (VUA) incorporating simultaneous PMFR with a single barbed suture [pontine VUA (P-VUA)] may facilitate continence recovery compared with the standard Van Velthoven anastomosis (ST-VUA). **Materials and Methods:** This prospective study included patients undergoing RARP between January 2021 and December 2023. Allocation was based on surgeon preference. UC was defined as the use of no pads or one dry safety pad per day and was assessed at 10, 30, 90, 180, and 365 days after catheter removal. Multivariable logistic regression was performed to evaluate factors associated with 30-day continence. Time to continence was additionally analyzed using Kaplan–Meier methods. **Results:** This prospective comparative study included 157 patients undergoing robot-assisted radical prostatectomy (RARP) between January 2021 and December 2023 (76 ST-VUA, 81 P-VUA). Baseline and pathological characteristics were comparable between groups. Catheterization time was significantly shorter in the P-VUA group (5.0 ± 1.1 vs. 6.7 ± 1.4 days, *p* < 0.001). Continence rates were higher in the P-VUA group at 10 days (72.8% vs. 55.3%, *p* = 0.03), 30 days (84.0% vs. 68.4%, *p* = 0.035), 90 days (92.6% vs. 76.3%, *p* = 0.007), 180 days (93.8% vs. 82.9%, *p* = 0.044), and 365 days (97.5% vs. 86.8%, *p* = 0.015). Kaplan–Meier analysis demonstrated a shorter time to continence in Group P (log-rank *p* = 0.0037). In multivariable analysis, P-VUA was independently associated with higher odds of 30-day continence (OR 6.38, 95% CI 2.08–19.63, *p* = 0.001). **Conclusions:** The study suggests that the P-VUA technique was associated with faster recovery of urinary continence compared with ST-VUA in this prospective, non-randomized cohort. These findings support the hypothesis that integrating anatomical reconstruction principles into the anastomotic step may enhance functional outcomes after RARP. However, the results should be interpreted with caution, given the study design and sample size, and require confirmation in larger, preferably randomized studies.

## 1. Introduction

Post-prostatectomy incontinence (PPI) for prostate cancer (PCa) is one of the most prevalent and distressing complications, profoundly affecting patients’ quality of life (QoL) [1,2,3,4]. With advancements in surgical techniques, the adoption of robot-assisted RP (RARP) has markedly improved outcomes, including better continence rates, compared with traditional laparoscopic and open RP approaches [4]. Despite these improvements, achieving earlier recovery of urinary continence (UC) remains one of the most important goals of contemporary prostate cancer surgery [5,6,7]. Furthermore, PPI is influenced by patient factors, including age, BMI, and Gleason score, as well as perioperative variables such as surgeon experience, nerve-sparing technique, lymph node dissection, surgical technique, and pelvic-floor rehabilitation [4,8,9]. However, to date, there is no clear definition of PPI, resulting in highly variable continence rates [10]. The most commonly employed method is the number of pads used within a 24-h period [11]. Various surgical strategies have been reported to facilitate UC recovery following RARP. These techniques include bladder neck preservation, nerve-sparing approaches, maintaining maximal urethral length, posterior musculofascial reconstruction (PMFR), anterior retropubic suspension, and bladder neck plication [12,13,14,15,16,17,18]. PMFR appears to be a particularly effective technique in achieving high UC rates while being associated with a lower rate of complications such as acute urinary retention (AUR), bladder neck stricture (BNS), and anastomotic leakage [10,19,20,21,22]. While PMFR has been previously associated with improved early UC (EUC) recovery, the potential benefit of integrating this step into a single running vesicourethral anastomosis (VUA) remains unclear. The present study aims to evaluate whether this modified “pontine” technique provides an incremental functional advantage over the standard Van Velthoven anastomosis without increasing surgical complexity or morbidity.

## 2. Materials and Methods

### 2.1. Study Design and Population

This prospective comparative study included consecutive male patients with histologically confirmed prostate cancer (PCa) who underwent robot-assisted radical prostatectomy (RARP) between January 2021 and December 2023 at a single tertiary referral center. All eligible patients were consecutively enrolled during the study period, and no additional screening exclusions were applied beyond the predefined criteria. All procedures were performed by two experienced robotic surgeons, each of whom had completed more than 150 RARPs prior to the study period, thus operating beyond the learning curve phase. Both surgeons performed both VUA techniques throughout the study period. Patients were allocated to one of two groups according to the VUA technique used: standard Van Velthoven VUA (ST-VUA) or modified “pontine” VUA with simultaneous PMFR (P-VUA). Allocation was based on surgeon preference and was not randomized. Both techniques were performed contemporaneously throughout the study period, and no temporal clustering of one technique over the other was observed. Exclusion criteria included pre-existing urinary incontinence, prior pelvic radiation therapy, previous prostatic surgery, or refusal to participate. All patients provided written informed consent for participation in the study and for the use of their anonymized clinical data for research purposes. The study was conducted in accordance with the Declaration of Helsinki and approved by the institutional review board and Ethics Committee of the Department of Medical and Surgical Sciences and Biotechnologies of Sapienza University in Rome (UROUNIVLT_1230/2020; approval date: 16 December 2020). Indications for extended pelvic lymph node dissection (ePLND) and nerve-sparing (NS) were determined according to European Association of Urology (EAU) [23] guidelines. To minimize potential confounding, perioperative management was standardized across both groups, including bladder neck preservation, urethral length preservation, posterior reconstruction, and postoperative care (catheter management and rehabilitation protocols). Clinical and demographic variables collected included age, body mass index (BMI), comorbidities [diabetes mellitus (DM), metabolic syndrome (MetS), cardiovascular disease, and use of antiplatelet/anticoagulant therapy], prostate-specific antigen (PSA), hemoglobin (Hb), biopsy ISUP grade, and prostate volume assessed by multiparametric magnetic resonance imaging (mpMRI). Perioperative variables included operative time, estimated blood loss, nerve-sparing (NS) approach, ePLND, length of hospital stay, and time to catheter removal. Final pathological outcomes included pTNM stage, surgical margin status, and pathological ISUP grade. Positive surgical margins were defined as tumor involvement exceeding 3 mm, in accordance with prior studies suggesting that margin length may have prognostic relevance [24].

### 2.2. Outcome Definition

The primary endpoint was early urinary continence. UC was defined as the use of no pads or one completely dry security pad per day (social continence). In our dataset, the use of a dry safety pad was considered clinically equivalent to no pad use, and therefore a separate strict pad-free definition (0 pads/day) was not available for analysis. UC was assessed at 10, 30, 90, 180, and 365 days after catheter removal, based on patient-reported pad use collected during standardized outpatient follow-up visits. No pad-weight testing or validated questionnaires were used. Patients who had already achieved continence at an earlier assessment and were subsequently not available for follow-up were classified as continent at later timepoints.

### 2.3. Surgical Technique

**Figure 1:**
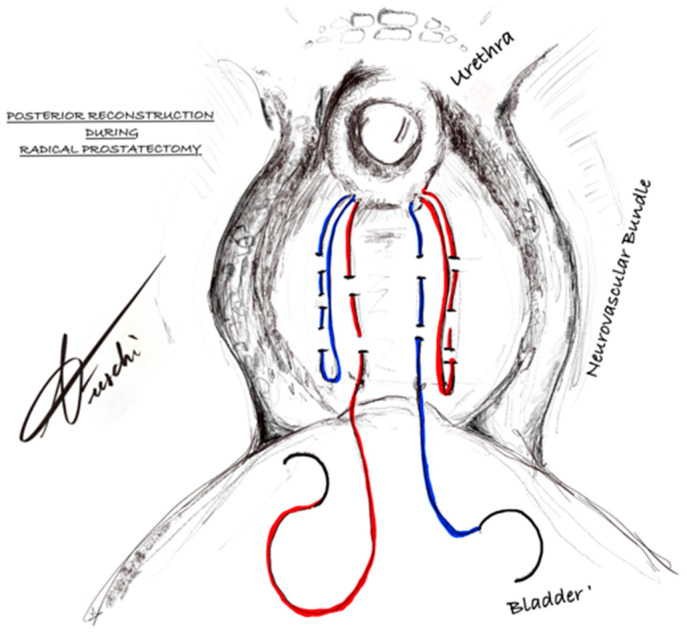
Schematic representation of the posterior musculofascial reconstruction during robot-assisted radical prostatectomy. The figure illustrates the reconstruction of the posterior layer between the bladder neck and urethra using a single running suture (red and blue), reinforcing the posterior support of the VUA.

The term “pontine” reflects both the bridging configuration created between the posterior musculofascial structures and the urethral stump, as well as the geographical origin of the technique at our center (Polo Pontino). PMFR was performed in both groups as part of standard surgical practice; however, the technique differed between groups. In Group ST, PMFR was performed as a conventional, separate step prior to completion of the VUA. In contrast, in Group P, PMFR was structurally integrated into the VUA using a single running barbed suture, resulting in a unified posterior support and anastomotic construct. The distinguishing feature of the proposed technique, therefore, lies in this integration of PMFR into the anastomotic step, rather than its performance as an independent maneuver. The first suture is placed with the needle oriented parallel to the Denonvilliers’ fascia and directed from right to left, at the level of the sub-urethral tissue (residual recto-urethralis muscle), taking care to avoid the urethra or any of its tissue. This pass is repeated using the contralateral needle, directed from left to right. With the needle now exiting the left sub-urethral tissue, a pass is made through the left lateral aspect of the Denonvilliers’ fascia, approximately 4 cm from the urethra, in a caudo-cranial direction, parallel to the rectum. The same maneuver is performed on the right lateral aspect using the right needle. Next, with the left needle (now exiting from the left lateral Denonvilliers’ fascia), a second pass is made through the sub-urethral tissue from left to right, ensuring that the suture lines do not cross. The needle is then passed through the right medial aspect of the Denonvilliers’ fascia, adjacent to the previous right-sided entry point, at which point the left needle becomes the new right-sided needle. The same step is repeated with the right needle, thereby making it the new left needle. At the end of this step, there are four passes through the Denonvilliers’ fascia and four through the sub-urethral tissue. Symmetric traction is then applied to the two suture threads exiting the Denonvilliers’ fascia, bringing the fascia into close apposition with the sub-urethral tissue. This PMFR creates a “urethral pull-through” effect, facilitating the subsequent VUA. The posterior anastomotic plate is then initiated from the urethral side, with the needle passing from inside the lumen outward. The left needle is passed at the 6–7 o’clock position, and the right needle at 6–5 o’clock. The procedure continues with the placement of two central sutures: one at 7 o’clock and one at 5 o’clock on the posterior bladder neck. It is important to include the mucosal layer in all sutures on both the urethral and bladder neck sides. Subsequently, two additional sutures are placed between the urethra and bladder: on the left at 8 and 9 o’clock, and on the right at 4 and 3 o’clock. After placing six urethral sutures, gentle traction is applied to the threads to complete the posterior plate. This configuration, with three passes per side, provides adequate reinforcement and helps prevent urethral tearing. Once the posterior plate is complete, an 18 Ch Foley catheter is advanced into the bladder. The anterior plate is then constructed with three sutures on the left and three on the right. At this stage, both suture threads exit from the urethra at the 12 o’clock position. The final pass is made through the anterior bladder wall. Once adequate tension is applied to ensure a watertight seal, the VUA is completed with three final knots tying the free ends. Figure 1 displays the anastomosis.

### 2.4. Statistical Analysis

Statistical analysis was performed using SPSS v.30 (IBM Corp., Chicago, IL, USA). Continuous variables were reported as mean ± standard deviation (SD) or median [interquartile range (IQR)], depending on the distribution assessed by the Shapiro–Wilk test. Categorical variables were presented as frequencies and percentages. Comparisons between groups were performed using the Student’s *t* test or Mann–Whitney *U* test for continuous variables and the χ^2^ test or Fisher’s exact test for categorical variables, as appropriate. A two-sided *p*-value < 0.05 was considered statistically significant. No formal sample size calculation was performed; therefore, the study should be considered exploratory.

Multivariable logistic regression analysis was performed to evaluate factors associated with urinary continence at 30 days after catheter removal, which was selected a priori as the primary early postoperative timepoint. The model included preoperative and intraoperative variables, namely age, BMI, prostate volume, biopsy ISUP grade, NS status, surgeon, and VUA technique. Postoperative pathological variables were not included. Given the limited number of events relative to the number of covariates included, the analysis should be considered exploratory and hypothesis-generating. Time to UC was additionally evaluated using Kaplan–Meier analysis. Event time was defined as the interval from catheter removal to the first scheduled follow-up visit at which continence was documented. Patients who did not achieve continence were censored at their last available follow-up. Patients lost to follow-up after already achieving continence were recorded as having experienced the event at the first timepoint at which continence was observed. Kaplan–Meier curves were compared using the log-rank test, and numbers at risk were reported below the x-axis.

## 3. Results

### 3.1. Baseline Characteristics

A total of 157 patients were included in the study. Of these, 76 patients underwent standard VUA (Group ST) and 81 received the pontine VUA anastomosis (Group P). Baseline characteristics were comparable between groups. The mean age was 62.68 ± 5.20 years in Group ST and 61.96 ± 4.88 years in Group P (*p* = 0.37). BMI was similar (26.88 ± 2.42 kg/m^2^ in Group ST vs. 26.73 ± 2.60 kg/m^2^ in Group P, *p* = 0.70), as was prostate volume (43.29 ± 13.87 mL vs. 43.96 ± 14.76 mL, *p* = 0.77) and PSA (8.20 ± 3.28 ng/mL vs. 8.10 ± 3.54 ng/mL, *p* = 0.85). The prevalence of DM was 14.5% in Group ST and 16.0% in Group P (*p* = 0.96), and MetS was reported in 22.4% and 18.5% of participants, respectively (*p* = 0.89). Preoperative Hb was 14.26 ± 1.10 g/dL in Group ST and 14.57 ± 1.36 g/dL in Group P (*p* = 0.10). ISUP grade at biopsy showed no statistically significant difference (*p* = 0.42). Table 1 summarizes the baseline characteristics.

### 3.2. Post-Operative Outcomes

Postoperative Hb was higher in Group P (13.03 ± 1.38 g/dL) than in Group ST (12.55 ± 1.07 g/dL, *p* = 0.058). The ΔHb was also lower in Group P (1.54 ± 0.70 g/dL) compared to Group ST (1.71 ± 0.76 g/dL, *p* = 0.063). EBL was similar between Group ST and Group P (315.46 ± 73.82 mL vs. 312.78 ± 71.24 mL, *p* = 0.81), as was operative time (153.7 ± 18.9 min vs. 158.9 ± 21.6 min, *p* = 0.088). Time to catheter removal was shorter in Group P (5.0 ± 1.1 days) than in Group ST (6.7 ± 1.4 days, *p* < 0.001). Length of hospital stay did not differ significantly (3.47 ± 1.19 days in Group ST vs. 3.42 ± 1.24 days in Group P, *p* = 0.69). The majority of patients received a nerve-sparing technique: in Group ST, 14.5% of patients did not receive a NS technique, while 31.6% and 53.9% received monolateral and had bilateral NS, respectively; in Group P, the rates were 19.8%, 30.9%, and 49.4%, respectively (*p* = 0.67). At final pathology, ISUP 3 increased in both groups: from 28.9% to 34.2% in Group ST and from 25.9% to 32.1% in Group P, reflecting pathological upgrading. The overall ISUP grade distribution at prostatectomy remained similar between groups (*p* = 0.55). Lymph node dissection was performed in 43.4% of Group ST and 45.7% of Group P (*p* = 0.90), with positive lymph nodes found in 5.26% and 6.2%, respectively (*p* = 0.74). Pathological staging was similar between groups (*p* = 0.93). In Group ST vs. Group P, the distribution was as follows: pT2a in 11.8% vs. 12.3%, pT2b in 39.5% vs. 39.5%, pT2c in 26.3% vs. 25.9%, pT3a in 18.4% vs. 19.8%, and pT3b in 1.3% vs. 2.5%. Positive surgical margins occurred in 9.2% of Group ST and 9.8% of Group P (*p* = 0.61). Anastomotic leak occurred in one patient in Group ST (1.31%) and in none in Group P (*p* = 0.48). AUR was observed in 2/76 patients (2.63%) in Group ST and 1/81 patients (1.23%) in Group P (*p* = 0.61). BNS was observed in one patient per group (1.31% in Group ST and 1.23% in Group P, *p* = 1). Post-operative outcomes are shown in Table 2.

### 3.3. Continence Outcomes

**Figure 2:**
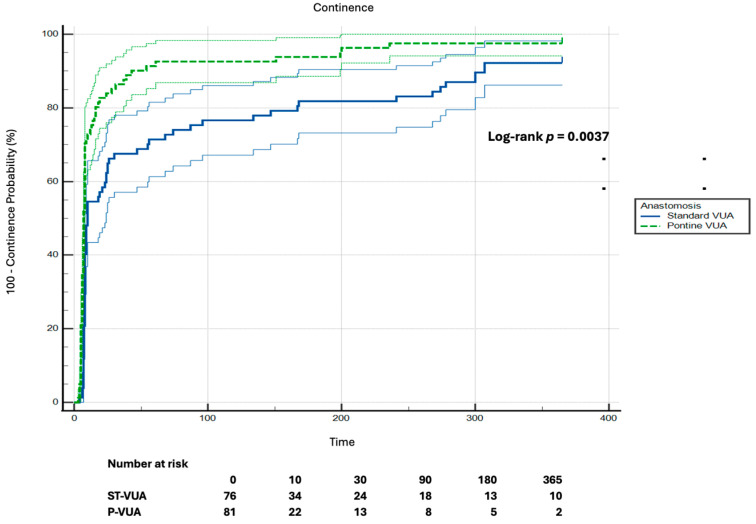
Kaplan–Meier analysis of time to UC stratified by anastomotic technique. Continence was defined as the use of no pads or one dry safety pad per day (log-rank *p* = 0.0037). Shaded areas represent 95% confidence intervals. Patients who achieved continence and were subsequently lost to follow-up were recorded as having experienced the event at the first timepoint at which continence was documented. Numbers at risk at predefined timepoints (0, 10, 30, 90, 180, and 365 days) are shown below the x-axis.

At 10 days post-catheter removal, 59/81 (72.8%) of patients in Group P were continent compared to 42/76 (55.3%) in Group ST (*p* = 0.03). At 30 days, UC was achieved in 68/81 (84.0%) and 52/76 (68.4%) in groups P-VUA and ST-VUA, respectively (*p* = 0.035). At 90 days, UC was achieved in 92.6% (75/81) of Group P and 76.3% of Group ST (58/76, *p* = 0.007). A total of 7 patients were lost after already achieving UC (Group P: *n* = 2 at 180 days and *n* = 3 at 365 days; Group ST: *n* = 1 at 180 days and *n* = 4 at 365 days). These patients were considered continent at subsequent timepoints. At 180 days, rates were 93.8% vs. 82.9% (Group P 76/81 vs. Group ST 63/76, *p* = 0.044), and at 365 days, 97.5% vs. 86.8% in groups P (79/81) and ST (66/76), respectively (*p* = 0.015). Kaplan–Meier analysis showed a shorter time to continence in Group P compared to Group ST (log-rank *p* = 0.0037). The number of patients at risk decreased over time in both groups (ST-VUA: 76 at baseline, 34 at 30 days, 10 at 365 days; P-VUA: 81 at baseline, 22 at 30 days, 2 at 365 days). The full numbers at risk are shown in Figure 2.

### 3.4. Multivariate Analysis

In multivariable logistic regression analysis including age, BMI, prostate volume, biopsy ISUP grade, nerve-sparing status, surgeon, and anastomotic technique, the P-VUA technique was associated with higher odds of urinary continence at 30 days compared to standard anastomosis (OR: 6.38, 95% CI: 2.08–19.63, *p* = 0.001). Higher biopsy ISUP grade was associated with lower odds of continence (OR: 0.47, 95% CI: 0.24–0.91, *p* = 0.02), while NS status was associated with higher odds of UC (OR: 9.64, 95% CI: 1.89–49.06, *p* = 0.006). Age showed a borderline association with continence (*p* = 0.082). BMI, prostate volume, and surgeon were not significantly associated with UC at 30 days. Given the limited number of events relative to the number of covariates included, these findings should be interpreted with caution. Table 3 displays multivariable analysis.

## 4. Discussion

The VUA represents one of the most important steps in RP, as it restores continuity between the bladder neck and the urethra. A precise, watertight VUA not only prevents urine leakage but also minimizes peri-anastomotic fibrosis, which can significantly impact UC recovery [25]. Indeed, evidence from open RP has shown that a continuous, well-sealed VUA is associated with reduced urine leak rates and faster return to UC compared to interrupted suturing [26]. PMFR aims to reapproximate and support the posterior aspect of the sphincter mechanism after prostate removal [27]. By reattaching Denonvilliers’ fascia and residual peri-urethral supporting tissues to the bladder neck or urethral stump, PMFR restores the anatomical support and increases the functional length of the urethra [28]. This additional support has been associated with earlier continence recovery in open surgery, and similar findings have also been reported in RARP series [19,29].

Notably, PMFR is a relatively simple modification that typically poses little risk of complications, such as strictures or leaks, and has become a widely adopted technique to enhance UC [19,25]. In this context, we hypothesized that optimizing the VUA technique by integrating a robust PMFR with a single suture would further improve UC outcomes after RARP. In our prospective comparison, the P-VUA technique was associated with earlier recovery of urinary continence compared with ST-VUA, suggesting that anatomical reconstruction may facilitate functional recovery. Patients who received the P-VUA showed higher UC rates across early postoperative timepoints. At 10 days post-catheter removal, 72.8% of Group P achieved UC compared to 55.3% of Group ST (*p* = 0.03). This advantage remained evident up to the 12-month follow-up (97.5% vs. 86.8%, *p* = 0.015), although the absolute difference between groups was smaller than in the early postoperative period. These findings suggest that although most patients eventually regained continence within a year, the P-VUA technique might lead to an earlier return to urinary control during the initial postoperative months when urinary incontinence has the greatest impact on QoL. Multivariable analysis was consistent with this observation, as the P-VUA technique was associated with higher odds of EUC recovery. However, the significance of this association should be interpreted with caution, given the width of the CI and the sample size. Nerve-sparing was associated with improved continence recovery in the multivariable analysis, which is consistent with its known role in preserving sphincteric function [17]. However, the study was not designed to evaluate differential effects across specific NS subgroups, and these findings should be interpreted cautiously. Our study contributes to an increasing body of research focused on reducing PPI through improvements in surgical technique. In addition to posterior support alone, total anatomical reconstruction (TAR) methods aim to restore both posterior and anterior support structures around the vesicourethral junction. TAR typically combines posterior reconstruction with an anterior reconstruction of the puboprostatic ligaments and tendinous arch of the pelvic fascia. The impact of TAR on early continence has been notable in several studies. For example, a multicenter randomized trial by Hurtes et al. [30] found significantly higher continence rates at 1 and 3 months with TAR compared to standard VUA. Another randomized study by Student et al. [31] reported that at 24 h post-catheter removal, 22% of patients were already continent compared to 6% in the standard VUA group. By 4 weeks, 62.5% of the TAR group was pad-free compared to 15% in the control group (*p* < 0.001). Higher EUC rates were reported in a series by Porpiglia et al. TAR achieved EUC rates of 78% at 7 days and 89% at 4 weeks [32]. The sustainable functional urethral reconstruction (SFUR) is another innovative technique that uses a tubularized bladder neck and reinforced peri-urethral support to mimic native anatomy [33]. In a 2021 series, continence rates at catheter removal were 34% with SFUR versus 4% with a standard anastomosis, increasing to 62% versus 28% at 4 weeks and 79% versus 63% at 3 months (all *p* < 0.05). Although study designs differ, posterior reconstruction, TAR, and SFUR have all been associated with earlier recovery of urinary continence. In line with these findings, our results suggest that a single continuous suture technique incorporating PMFR may be associated with improved early continence outcomes, with results broadly comparable to those reported for more extensive reconstruction techniques. Others have also described this single-suture approach; for example, Flammia et al. reported a “single-knot, single running suture” VUA with PMFR, demonstrating the feasibility of combining reconstruction and anastomosis within a single suture line [22]. The current findings should be viewed as hypothesis-generating. While the modified technique was associated with better EUC recovery, the incremental nature of this change compared to established reconstruction methods should be highlighted. Given the non-randomized design and surgeon-preference allocation, residual confounding cannot be excluded; therefore, definitive claims of superiority cannot be made. Further comparative studies are needed to determine whether this approach provides a clinically meaningful benefit.

## 5. Limitations

This study has several limitations that should be acknowledged. First, it was conducted at a single high-volume center by experienced robotic surgeons, which may limit the extent to which the findings apply to lower-volume settings or to surgeons earlier in their learning curve. Second, the study design was non-randomized, and allocation to the surgical technique was based on surgeon preference. Although baseline characteristics were broadly comparable between groups and perioperative management was standardized, residual confounding and selection bias cannot be excluded. Third, the sample size was small, especially for less common outcomes, which may have affected the precision of some estimates. This is evident from the wide CI observed in the multivariable analyses, so the results should be interpreted with caution. Fourth, UC was measured using patient-reported pad use, without validated questionnaires or objective pad-weight testing. While this reflects typical clinical practice, it may introduce reporting bias and limit comparison with studies that use standardized outcome measures. Additionally, baseline urinary function was not thoroughly characterized. Fifth, continence was assessed at set follow-up points rather than continuously, and time-to-event analyses relied on interval-censored data, potentially reducing temporal accuracy. Finally, this study mainly focused on functional outcomes. Other important endpoints, such as sexual function, patient-reported QoL, and cost considerations, were not examined. Furthermore, no long-term cancer outcomes were assessed. Overall, the findings should be viewed as exploratory and hypothesis-generating. Further validation through larger, prospective, and ideally randomized multicenter studies is necessary.

## 6. Conclusions

In conclusion, the “pontine” VUA with simultaneous PMFR represents a feasible modification of standard RARP. In this prospective cohort, the technique was associated with earlier UC recovery compared with the standard approach. By integrating posterior support into the anastomotic step, this approach builds on established continence-preserving principles while streamlining the technique. While most patients in both groups ultimately achieved UC over time, the modified technique may facilitate an earlier return to urinary control. No clear differences in perioperative complications were observed; however, the study was not powered to detect differences in rare adverse events or long-term oncologic outcomes. These findings should be interpreted with caution, given the non-randomized design and the potential for residual confounding. Therefore, the results should be considered hypothesis-generating. Further validation in larger, multicenter, and randomized studies is warranted to confirm the clinical relevance of this approach and to better define its role in contemporary RARP.

## Figures and Tables

**Table 1:** Baseline demographic and preoperative clinical characteristics of patients undergoing robot-assisted radical prostatectomy with standard vesicourethral anastomosis (ST-VUA) and pontine vesicourethral anastomosis (P-VUA).

Variable	Group ST (76 Patients)	Group P (81 Patients)	*p*
Age, y ± SD	62.68 ± 5.20	61.96 ± 4.88	0.37
Prostate Volume, mL ± SD	43.29 ± 13.87	43.96 ± 14.76	0.77
BMI, kg/m^2^ ± SD	26.88 ± 2.42	26.73 ± 2.60	0.7
DM, *n* (%)	11/76 (14.5)	13/81 (16.0)	0.96
MetS, *n* (%)	17/76 (22.4)	15/81 (18.5)	0.89
PSA, ng/mL ± SD	8.20 ± 3.28	8.10 ± 3.54	0.85
Preoperative Hb, g/dL ± SD	14.26 ± 1.1	14.57 ± 1.36	0.1
*Pathological Grade At Biopsy*, *n* (%)			0.42
ISUP 1	6/76 (7.9)	2/81 (2.5)	
ISUP 2	37/76 (48.7)	41/81 (50.6)	
ISUP 3	22/76 (28.9)	21/81 (25.9)	
ISUP 4	8/76 (10.5)	10/81 (12.3)	
ISUP 5	3/76 (3.9)	7/81 (8.6)	

**Table 2:** Intraoperative, pathological, and postoperative outcomes of patients undergoing RARP with standard vesicourethral anastomosis (ST-VUA) and pontine vesicourethral anastomosis (P-VUA). Data include operative variables, pathological grade and stage, nerve-sparing status, perioperative complications, and continence recovery rates.

Variable	Group ST (76 Patients)	Group P (81 Patients)	*p*
Operative Time, min ± SD	153.7 ± 18.9	158.9 ± 21.6	0.088
EBL, mL ± SD	315.46 ± 73.82	312.78 ± 71.24	0.81
Postoperative Hb, g/dL ± SD	12.55 ± 1.07	13.03 ± 1.38	0.058
ΔHb, g/dL ± SD	1.71 ± 0.76	1.54 ± 0.7	0.063
*Pathological Grade At Prostatectomy*, *n* (%)			0.55
ISUP 1	5/76 (6.6)	2/81 (2.5)	
ISUP 2	34/76 (44.7)	36/81 (44.4)	
ISUP 3	26/76 (34.2)	26/81 (32.1)	
ISUP 4	8/76 (10.5)	10/81 (12.3)	
ISUP 5	3/76 (3.9)	7/81 (8.6)	
*Nerve Sparing*, *n* (%)			0.67
No	11/76 (14.5)	16/81 (19.8)	
Monolateral	24/76 (31.6)	25/81 (30.9)	
Bilateral	41/76 (53.9)	40/81 (49.4)	
ePLND, *n* (%)	33/76 (43.4)	37/81 (45.7)	0.9
*Pathological Stage*, *n* (%)			0.93
T2a	9/76 (11.8)	10/81 (12.3)	
T2b	30/76 (39.5)	32/81 (39.5)	
T2c	20/76 (26.3)	21/81 (25.9)	
T3a	14/76 (18.4)	16/81 (19.8)	
T3b	1/76 (1.3)	2/81 (2.5)	
Positive Lymphnodes, *n* (%)	4/76 (5.26)	5/81 (6.2)	0.74
Positive Surgical Margin (>3 mm), *n* (%)	7/76 (9.2)	8/81 (9.8)	0.61
*Postoperative Outcomes*			
Length of Hospital Stay, Days ± SD	3.47 ± 1.19	3.42 ± 1.24	0.69
Time To Catheter Removal, Days ± SD	6.7 ± 1.4	5.0 ± 1.1	<0.001
Anastomotic Leakage, *n* (%)	1 (1.31)	0	0.48
Acute Urinary Retention, *n* (%)	2 (2.63)	1 (1.23)	0.61
BNS, *n* (%)	1 (1.31)	1 (1.23)	1
*Continence Outcomes*, *n* (%)			
10 Days	42/76 (55.3)	59/81 (72.8)	0.03
30 Days	52/76 (68.4)	68/81 (84.0)	0.035
90 Days	58/76 (76.3)	75/81 (92.6)	0.007
180 Days	63/76 (82.9)	76/81 (93.8)	0.044
365 Days	66/76 (86.8)	79/81 (97.5)	0.015

**Table 3:** Multivariable logistic regression analysis of predictors of urinary continence at 30 days after robot-assisted radical prostatectomy. Variables included anastomotic technique (P-VUA vs. ST-VUA), age, prostate volume, body mass index (BMI), biopsy ISUP grade, nerve-sparing status, and surgeon. Odds ratios (ORs), 95% confidence intervals (CIs), and *p*-values are reported.

Variable	OR	CI 95% (Lower–Upper)	*p*
P-VUA	6.38	2.08–19.63	0.001
Age	1.09	0.99–1.2	0.082
Prostate volume	1.02	0.99–1.06	0.21
BMI	1.08	0.86–1.35	0.52
ISUP at biopsy	0.47	0.24–0.91	0.02
Surgeon	1.15	0.45–0.91	0.77
Nerve sparing	9.64	1.89–49.06	0.006

## Data Availability

Data are unavailable due to privacy or ethical restrictions imposed by our institution.

## Abbreviations

The following abbreviations are used in this manuscript:

AUR	Acute Urinary Retention
BMI	Body Mass Index
BNS	Bladder Neck Stricture
CI	Confidence Interval
DM	Diabetes Mellitus
ePLND	Extended Pelvic Lymph Node Dissection
EUC	Early Urinary Continence
Hb	Hemoglobin
ISUP	International Society of Urological Pathology
MetS	Metabolic Syndrome
mpMRI	Multiparametric Magnetic Resonance Imaging
NS	Nerve Sparing
OR	Odds Ratio
PCa	Prostate Cancer
PMFR	Posterior Musculofascial Reconstruction
PSA	Prostate-Specific Antigen
PPI	Post-Prostatectomy Incontinence
P-VUA	Pontine Vesicourethral Anastomosis
QoL	Quality of Life
RARP	Robot-Assisted Radical Prostatectomy
RP	Radical Prostatectomy
SD	Standard Deviation
ST-VUA	Standard Vesicourethral Anastomosis (Van Velthoven)
TAR	Total Anatomical Reconstruction
UC	Urinary Continence
UI	Urinary Incontinence
VUA	Vesicourethral Anastomosis
